# Micro-Halocline Enabled Nutrient Recycling May Explain Extreme *Azolla* Event in the Eocene Arctic Ocean

**DOI:** 10.1371/journal.pone.0050159

**Published:** 2012-11-16

**Authors:** Monique M. L. van Kempen, Alfons J. P. Smolders, Leon P. M. Lamers, Jan G. M. Roelofs

**Affiliations:** 1 Department of Aquatic Ecology and Environmental Biology, Faculty of Science, Institute for Water and Wetland Research, Radboud University, Nijmegen, The Netherlands; 2 B-WARE Research Centre, Nijmegen, The Netherlands; The Australian National University, Australia

## Abstract

In order to understand the physicochemical mechanisms that could explain the massive growth of *Azolla arctica* in the Eocene Arctic Ocean, we carried out a laboratory experiment in which we studied the interacting effects of rain and wind on the development of salinity stratification, both in the presence and in the absence of a dense *Azolla* cover. Additionally, we carried out a mesocosm experiment to get a better understanding of the nutrient cycling within and beneath a dense *Azolla* cover in both freshwater and brackish water environments. Here we show that *Azolla* is able to create a windproof, small-scale salinity gradient in brackish waters, which allows for efficient recycling of nutrients. We suggest that this mechanism ensures the maintenance of a large standing biomass in which additional input of nutrients ultimately result in a further expansion of an *Azolla* cover. As such, it may not only explain the extent of the *Azolla* event during the Eocene, but also the absence of intact vegetative *Azolla* remains and the relatively low burial efficiency of organic carbon during this interval.

## Introduction

*Azolla* is a pleustonic freshwater fern known from temperate, tropical and subtropical regions all over the world. Distinctive to *Azolla* is that it encloses a permanent endosymbiotic prokaryotic community inside its leaf cavities [1]. The most notable member of this community is the nitrogen fixing cyanobacterium *Anabaena azollae* which is able to meet the entire nitrogen demand of the symbiosis, as long as *Azolla* provides it with a fixed source of carbon [2], [3].

Fossil *Azolla* species have been recognised in freshwater deposits from the mid to late Cretaceous onward. They can be found as complete plants and/or as megaspores and microspores, the latter often clumped together in so called massulae [4], [5]. More recently, high abundances of morphologically intact fossil microspores and megaspores of *Azolla arctica* [6] were found in mid Eocene (∼48.5 Ma) marine sediments [7]. These were recovered from the Lomonosov ridge in the central Arctic Ocean during the Arctic Coring Expedition of the Integrated Ocean Drilling Program. Many of the encountered mature megaspores in these marine sediments had microspore massulae attached indicating that *Azolla arctica* grew and reproduced in situ in the Arctic basin during the middle Eocene when this area had a relatively warm climate [6], [7].

The sediments representing the Eocene *Azolla* event, which lasted at least 800 000 years [7], were found to be laminated and the observed cyclicity of spore abundances appeared to be strongly related to changes in obliquity and precession cycles [8]. Such orbital processes are known to affect pole-ward atmospheric heat and moisture transport [9]. Indeed, it has been computed that the warm greenhouse conditions of the Eocene period induced an intensified hydrological cycle with precipitation exceeding evaporation at high latitudes [10], [11]. In combination with the relatively isolated geography of the Arctic Ocean during the Eocene [12], this enhanced freshwater input (by the precipitation itself and/ or via river discharge) frequently resulted in a highly stratified Arctic Ocean with a relatively freshwater layer (1–6 %) on top of a more saline (15–21%), occasionally anoxic deepwater layer [7], [13], [14].

Such variations in salinity are also well known from present day estuaries where the vertical salinity distribution, which may evolve as a consequence of freshwater input by rivers, is often characterized by a very steep salinity gradient at the interface of two water layers differing in density. When such a halocline is formed, it often hampers the mixing between water layers, although it cannot entirely prevent salinity from influencing the top water layers along the estuary, due to the transport of incoming deep saline water and the influence of wind and wave action on the circulation of the water column [15]. In the Eocene Arctic Ocean the halocline apparently was very strong during very long periods and the surface waters were frequently fresh or at least brackish enough to allow for the growth of *Azolla arctica* [7], [13], [14].

Beside relatively fresh water conditions there also must have been a sufficiently large influx and availability of nutrients in order to be able to explain the massive growth of *Azolla* in the Eocene Arctic Ocean. There is strong evidence that dinitrogen fixation was already a persistent feature in the Eocene Arctic ocean [16], [17]. Therefore, the growth of *Azolla arctica* was presumably not limited by nitrogen, but by phosphorus.

**Figure 1:**
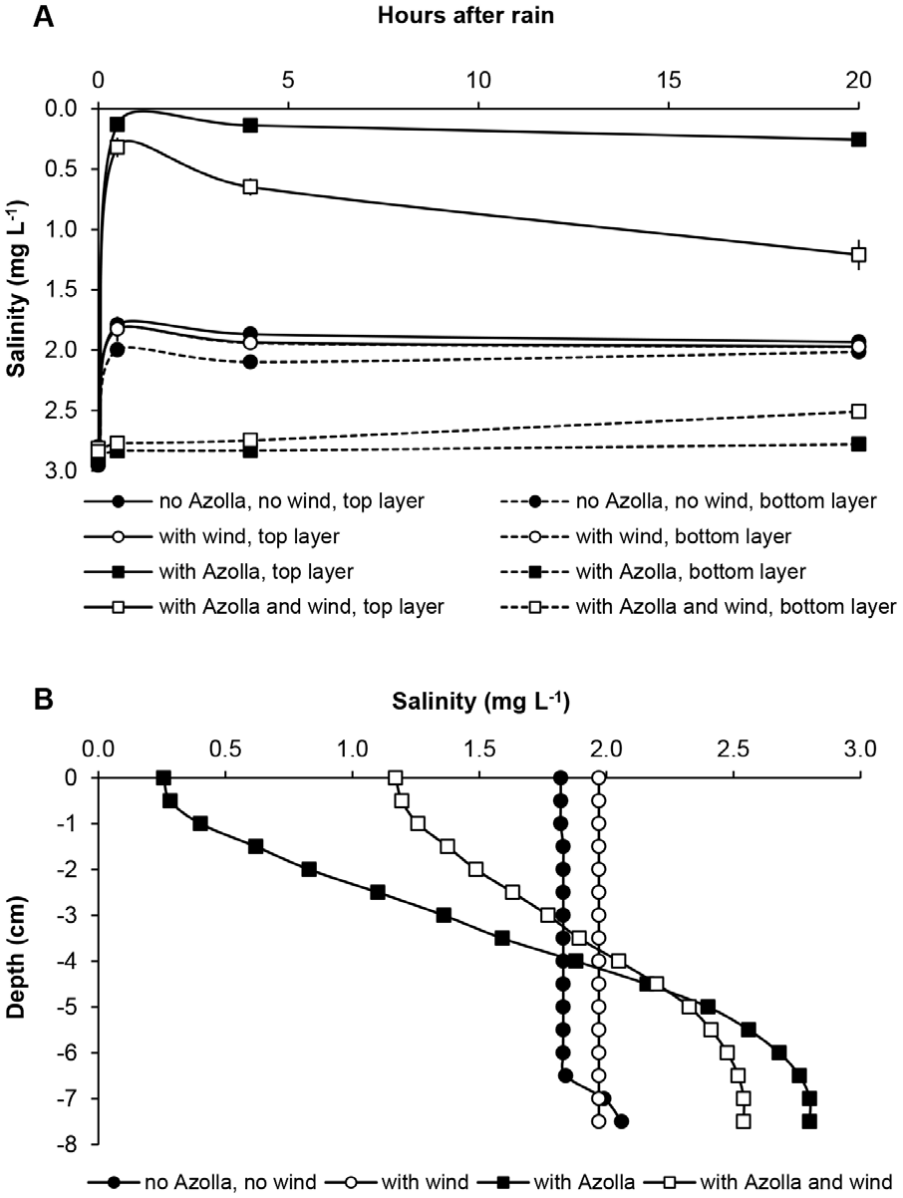
Interacting effects of *Azolla*, rain and wind on salinity stratification. A ) Salinity (mg L^−1^ ± standard error) in the top water layers (solid lines) and in the bottom water layers (dotted lines) of the beakers in the absence of *Azolla* (rounds), in the presence of *Azolla* (squares), with no influence of wind (closed figures) or with influence of wind (open figures) hours after the rain event. **B**) Salinity (mg L^−1^) profiles in the beakers 20 hours after the rain event.

Present day *Azolla* species show a high phosphorus demand and typically occur in relatively shallow water bodies [18], [19]. It has been shown that high phosphate concentrations of over 75 µM may still stimulate *Azolla* growth [20], [21], [22]. Since *Azolla* is only able to utilize the dissolved inorganic phosphate in the surface water, its growth is often limited by the release of phosphate from the sediment [18]. In open waters this often is too slow to meet its requirements, but the presence of a dense cover of *Azolla* frequently renders the surface water anoxic [23], [24], which in turn may enhance the phosphate release from the sediment [25], [26]. In shallow water bodies, where there is little dilution, such a reductive solubility of phosphate can considerably increase its availability in the surface water.

In the Eocene Arctic Ocean dissolved inorganic phosphate in the surface water originated from the release from sediments within and adjoining the Arctic Ocean, and/or from the inflow of rivers. Given that the basin was relatively deep and highly stratified at the time of the extensive *Azolla* blooms, it seems unlikely that much of the sediment derived phosphate within the Arctic Ocean became available to *Azolla arctica*. In stratified lakes it is well known that the presence of a halocline often results in a hypolimnetic nutrient accumulation [15], [26]. If this were also the case in the Eocene Arctic Ocean, this would mean that the growth of *Azolla arctica* was largely dependent on the release of phosphate from shallow sediments adjoining the Arctic Ocean and especially on the phosphate input via rivers. Therefore, the fact that *Azolla arctica* nevertheless was able to disperse all the way to the central Arctic ocean, can only point to an enormous expansion of the *Azolla* from the coastal regions and a probable mechanism for efficient recycling of nutrients in the surface water layer of the open ocean.

**Figure 2:**
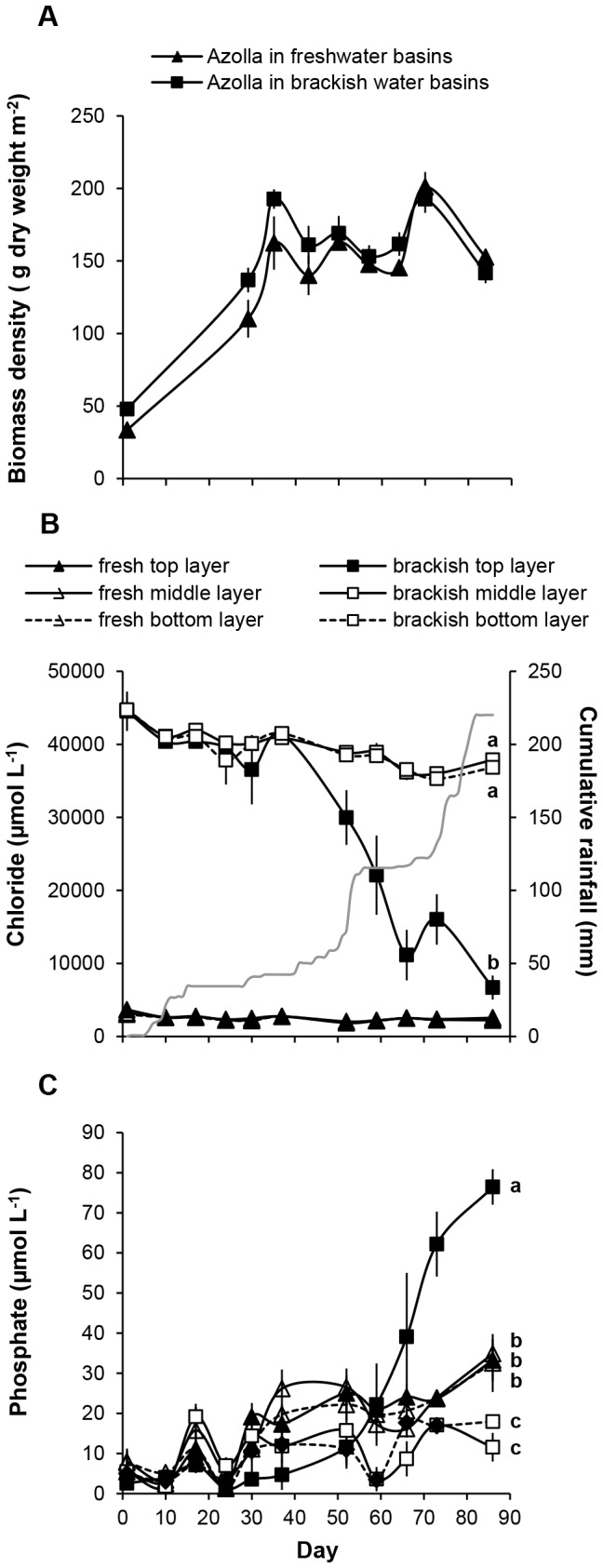
Mesocosm experiment. **A**) Development of the biomass density of *Azolla filiculoides* (g dry weight m^−2^ ± standard error) grown in freshwater or brackish water basins. **2B**) Chloride concentrations and **2C**) phosphate concentrations (µM ± standard error) in the top, middle and bottom water layers of the freshwater and brackish water basins during the mesocosm experiment. Significant differences between water layers are indicated by different letters. The cumulative amount of rainfall during the experiment (mm) is shown on the right axis in figure **2B**.

Here, we studied the effect of the presence of *Azolla* on the development of a small-scale salinity gradient in slightly brackish waters in order to empirically test the hypothesis that the development of such a small-scale halocline facilitates nutrient recycling within a dense *Azolla* cover by trapping the nutrients that are lost from the decomposition of dead plant material within the top surface water layer. First, we carried out a laboratory experiment in which we studied the interacting effects of rain and wind on the development of salinity stratification, both in the presence and in the absence of a dense *Azolla* cover. Next we carried out a mesocosm experiment in order to get a better understanding of the nutrient cycling within and beneath a dense *Azolla* cover in both freshwater and brackish water environments.

**Figure 3:**
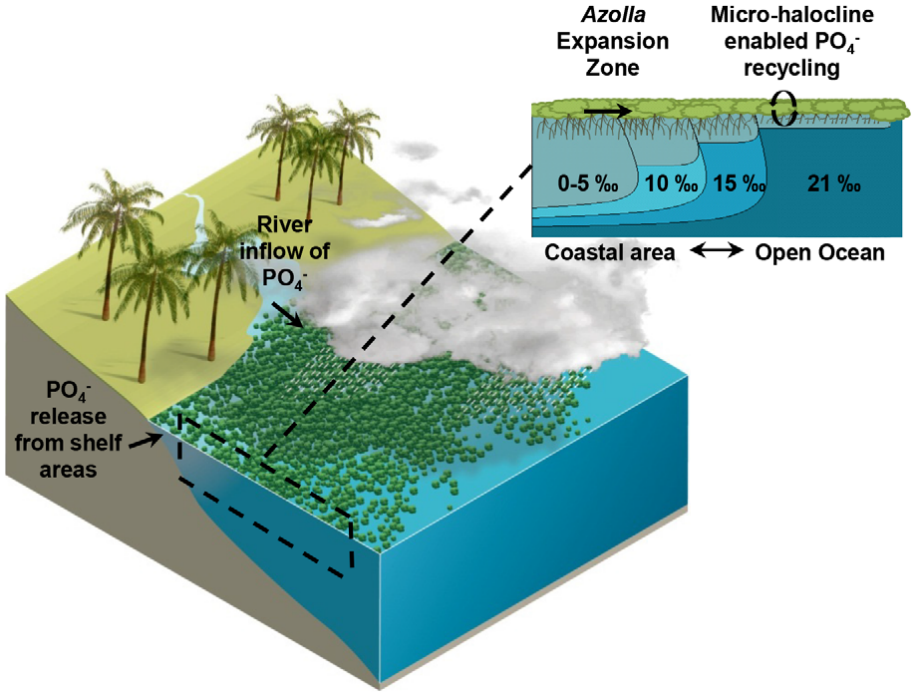
Conceptual model showing how *Azolla arctica* may have colonized the Eocene Arctic Ocean using phosphate sources from coastal areas for expansion to the open ocean where small-scale salinity stratification allows for efficient recycling of nutrients to sustain the standing biomass.

## Materials and Methods

### Species

We used *Azolla filiculoides*, which is the northernmost occurring *Azolla* species. It is widely distributed, including the subtropical and tropical regions of the world [27]. In the Netherlands, it mostly grows in the western parts of the country where the maritime climate tempers winter periods, but it is increasingly expanding its distribution eastward.

### Interacting effects of Azolla, rain and wind on salinity stratification

Sixteen 800 mL beakers were filled with 600 ml of a nutrient solution containing 1.5 mM NaHCO_3_, 0.5 mM KCl, 0.5 mM MgCl_2_6H_2_O, 0.025 mM Fe-EDTA, 0.025 mM NaH_2_PO_4_H_2_O, 0.001 mM CuSO_4_5H_2_O, 0.02 mM MnCl_2_4H_2_O, 0.01 mM ZnSO_4_7H_2_O, 0.003 mM Na_2_MoO_4_2H_2_O, 0.02 mM H_3_BO_3_ and 0.004 mM CoCl_2_6H_2_O (Sigma-Aldrich, Zwijndrecht, Netherlands). The composition of this growth medium was based on the results obtained in a field survey by de Lyon and Roelofs [28], who studied the distribution of *A. filiculoides*, among other aquatic plants in the Netherlands, in relation to water quality and other biogeochemical parameters. The final salinity of the nutrient solution was set to 3% by adding artificial sea salt (Tropic Marin, Wartenberg, Germany) and was determined by measuring the total dissolved salt concentration using a multi meter (18.52.SA multimeter, 18.56 Flow-through cell Compact, Eijkelkamp Agrisearch Equipment, Giesbeek, the Netherlands).

The surface waters of eight of the beakers were completely covered with 20 g fresh *A. filiculoides*, which had been cultivated in the laboratory prior to the experiment for approximately 8 weeks. Four of the covered and four of the non-covered beakers were subjected to wind influences. Wind was simulated by placing tubes diagonally above the *Azolla* or water surface from which air escaped at a speed of approximately 11.2 m s^−1^ (Windmaster 2, Kaindl Electronics, Rohrbach, Germany). Next, we subjected all sixteen beakers to a rain event which was simulated by sprinkling approximately 30 mm of demineralized water over the beakers from a distance of ∼1 meter using a watering can. The salinity of the nutrient solution was measured at the surface and at the bottom of the beakers at 0, 0.5, 4 and 20 hours after the rain event. At the end of the experiment (20 hours after the rain event) a complete salinity profile was determined by measuring the total dissolved salt concentration in the nutrient solution every 0.5 cm from the surface to the bottom of the beakers. The electrode was kept steady using a double column height dial gauge (Microscale measurements, The Hague, The Netherlands).

### Mesocosm experiment

In the Netherlands *A. filiculoides* typically grows from late summer to early winter and the mesocosm experiment was therefore conducted from mid September to early December in basins outside the experimental greenhouse of the Radboud University Nijmegen (N51°82′29”; E5°87′16”). The plants used for inoculation had been collected from a ditch near an arable land in Elst, The Netherlands (N51°55′48”; E5°50′6”). No specific permits were required for this. At the time of collecting the *Azolla* the location was not privately owned or protected in any way.

Six semi-enclosed basins, with a depth of 92 cm and a radius of 185 cm (∼2500 liter), were filled with tap water and different chemicals were added to establish a nutrient solution with a composition similar to what was used in the laboratory experiment. In three of the six basins, however, artificial sea salt was not included. To avoid the influence of micro-climate differences a complete random design was used. The experiment started with the inoculation of ∼2.7 kg (1 kg m^−2^) fresh *A. filiculoides* to all of the basins. To prevent the depletion of phosphorus, iron and other micronutrients, their concentrations were restored at days 24 and 37. The experiment lasted 86 days. The amount of rainfall was monitored during the experiment, and from the moment that the basins were fully covered with *A. filiculoides* we measured dissolved oxygen concentrations and temperatures at the surface and the bottom of the basins using an YSI multiparameter probe (YSI Incorporated, Yellow Springs, USA).

Biomass sub-samples were taken at regular time intervals from the moment the basins were fully covered with *A. filiculoides* using a square sieve of 25 cm^2^. The harvested plants were washed with running demineralized water for half a minute and blotted dry with tissue paper, after which their fresh weight was determined. After drying at 70°C for 48 hours, dry weight was determined.

The nutrient solution in the basins was sampled weekly at the surface (top 5 cm), in the middle (45–50 cm at depth) and at the bottom (87–92 cm at depth). The sampling was carried out using 30 mL vacuum flasks connected to ceramic soil moisture samplers (Eijkelkamp Agrisearch Equipment, Giesbeek, the Netherlands). Concentrations of PO_4_^3−^ and Cl^−^ were measured colorimetrically and concentrations of K^+^ and Na^+^ were measured flame photometrically using Auto Analyzer systems (Bran & Luebbe, Norderstedt, Germany). Total Ca, Mg and S concentrations were determined using an inductively-coupled plasma emission spectrophotometer (ICP-OES, model IRIS Intrepid II XDL, Thermo Electron Corporation, Franklin, MA).

### Statistics

We used Mixed Linear Models in SPSS 17.0 for Windows (SPSS Inc., Chicago, IL, U.S.A.) for statistical analyses. For the laboratory experiment we nested time as a repeated co-variable of the beakers and chose heterogeneous auto-regressive as a covariance type. We entered water layer (top or bottom), plant (with or without *Azolla*) and wind (wind or no wind) as fixed factors. For the analysis of the biomass data in the mesocosm experiment we used the same model except that we chose auto-regressive as a covariance type and entered treatment (fresh or brackish) as a fixed factor. The chloride concentrations in the brackish water basins were analyzed separately because salinity levels in the fresh and brackish water basins obviously were different. We only focussed on the concentrations in the different water layers that were measured from day 37 onward. We nested time as repeated co-variable of the basins and chose heterogeneous auto-regressive as a covariance type. The differences between the water layers in the brackish water basins were determined using Bonferroni post hoc analysis. We ran a univariate ANOVA on the chloride concentrations in the top water layers of the fresh and brackish water basins to see if they had become comparable at the end of the experiment. The dissolved oxygen concentrations and phosphorus concentrations were analyzed in a similar way as the chloride concentration, only now the water layers of the fresh water basins were included in the analyses, which voided the need to perform a separate univariate ANOVA on the data at the end of the experiment. In the data analysis of the phosphorus concentrations we used unstructured variations as a covariance type.

## Results

### Interacting effects of Azolla, rain and wind on salinity stratification

We found that the salinity of the top and bottom water layers became significantly different after the rain event (F_1_ = 3053.573, *p*<0.001) and that the presence or absence of *Azolla* (F_1_ = 262.963, *p*<0.001) or wind (F_1_ = 12.409, *p*<0.01) had a significant effect. The results of the laboratory experiment further showed that the presence of the *Azolla* cover significantly enhanced salinity stratification (F_1_ = 2500.946, *p*<0.001), whereas wind influences significantly decreased salinity stratification (F_1_ = 76.587, *p*<0.001). The presence of the *Azolla* cover significantly hampered the influence of wind mixing the water column (F_1_ = 30.795, *p*<0.001). In addition, we found a significant interaction between the salinity of the different water layers, the presence or absence of *Azolla* and the presence or absence of wind (F_1_ = 13.876, *p*<0.001) (Fig. 1A).

Also from the salinity profiles, which were taken 20 hours after the rain event, it became clear that the salinity gradient was steepest in the treatment with *Azolla* when there was no wind, followed by the treatment with *Azolla* and wind. In the treatment without *Azolla* and without wind, only a small gradient was left at the bottom of the beakers, whereas in the treatment without *Azolla* but with wind no gradient was found (Fig. 1B).

### Mesocosm experiment

During the first ∼35 days of the mesocosm experiment the biomass in the basins increased. Biomass densities of *Azolla* in the freshwater and brackish water treatment did not differ (F_1_ = 2.817, p>0.05) (Fig. 2A). The *Azolla* cover, including roots, was approximately 5–7 cm thick and the roots were 3–5 cm long. At the end of the experiment part of the biomass started to decompose due to decreasing temperatures. The minimum air temperature at 10 cm above the ground occasionally fell below the freezing point, but the water temperature in the basins never became lower than 4.4°C (Fig. S1).

In figure 2B the chloride concentration in the different water layers is shown as an indicator for the observed ionic salinity changes in the fresh and brackish water basins. The Ca^2+^, Mg^2+^, Na^+^, K^+^, SO_4_^−^ concentrations showed similar patterns (Fig. S2). At the start of the experiment, Cl^-^ concentrations were evenly distributed throughout the water column, both in the fresh and brackish water basins. However, after ∼37 days the top water layer in the brackish water basins became significantly fresher than the middle and the bottom water layers (F_2_ = 12.662, *p*<0.01). The freshening of the top water layer in the brackish basins started at a day that had more than 25 mm of rain (Fig. 2B). At the end of the experiment the chloride concentration in the top water layer of the brackish water basins had become similar to the chloride concentration in the top water layer of the fresh water basins (F_1_ = 7.143, p>0.05) (Fig. 2B). This salinity stratification in the brackish water basins also became clear from the oxygen concentrations in the different water layers that, as opposed to the different water layers in the freshwater basins, showed a clear discontinuity between the top surface water layer and the bottom water layer (Fig. S3).

At the end of the experiment the dissolved inorganic phosphate concentrations in the basins started to increase due to the decomposition of dead biomass. The phosphate in the freshwater basins distributed equally throughout the water column, but in the brackish water basins the phosphate strongly accumulated in the top water layer (F_5_ = 356.132, *p*<0.001) (Fig. 2C).

## Discussion

The results of the present research clearly show that when *Azolla* is growing in a brackish water environment it is able to generate small-scale salinity stratification (micro-halocline at approximately 5–7 cm) by muffling the force of the incoming rain and trapping the rainwater in between its biomass. For the Eocene Arctic basin it has been shown that salinity stratification was preserved during very long periods and that it was especially strong at times when spore abundances of *Azolla arctica* were very high [7], [8], [13]. The results of this research show that it is plausible that the presence of *Azolla arctica* in the Eocene Arctic basin contributed to the development of small-scale salinity gradients within the gradient that had already been established due to the enhanced freshwater input via rain and rivers [10], [11], not only by trapping rainwater, but also by diminishing the influence of wind action on the mixing of water layers.

Present day *Azolla* species have been shown to be able to grow at salinities up to 7–10% [29], [30]. In addition, at even higher salt concentrations *A. filiculoides* may avoid salinity stress by shedding of roots as this temporarily prevents the development of ionic imbalances within the plant tissue (MML van Kempen, unpubl.). With regard to the Eocene era, such a protective mechanism, avoiding salinity stress at temporarily high salt concentrations, might have facilitated the expansion of *Azolla arctica* further away from the coast by helping it to survive sudden salinity changes and providing it with the opportunity to swiftly generate its own freshwater environment before regenerating its roots (Fig. 3).

In the mesocosm, decreasing temperatures resulted in a net mineralization of organic matter towards the end of the experiment. Evidently, the mineralization of the biomass mainly occurred in the surface water layer within the *Azolla* cover and, judging on the total dissolved phosphate concentration, was equally high in the fresh and brackish water basins. In the brackish water basins the nutrients that were released upon mineralization accumulated mostly in the top water layer, whereas in the freshwater basins these nutrients became distributed more equally throughout the water column. Most probably this was due to the salinity stratification in the brackish water basins, which can function as a chemical barrier [15]. Usually a halocline prevents phosphorus that is released from decomposing organic matter at the bottom of water bodies from reaching the water surface [15], [26]. In a floating *Azolla* mat, however, salinity stratification apparently works in a reverse way by preventing phosphorus that is released from biomass mineralization in the top surface water layer from becoming dispersed throughout the entire water body. Obviously, this strongly increases the potential recycling of nutrients within the system (Fig. 3).

*Azolla* is able to grow at low phosphate concentrations, but adding phosphate will almost always result in higher growth rates, even at very high concentrations [20], [22]. From the current research we conclude that *Azolla* acts as an ecosystem engineer being able to create a small scale salinity gradient within brackish waters in which potential salt stress is reduced and the efficient recycling of nutrients permits the maintenance of the standing biomass (Fig. 3). As such, we might presume that additional inputs of phosphate will ultimately result in a further expansion of an *Azolla* cover if other environmental factors are favourable and space is not limiting. With regard to the Eocene the release of phosphate from flooded shelf areas adjoining the Arctic basin and the entering of phosphate via river discharge, together with the micro-halocline enabled nutrient recycling at long distances from the coast, may have facilitated the enormous expansion of *Azolla arctica* from the coastal areas to the open ocean. Consequentially, our results may help to understand the extent of the Eocene *Azolla* event and may explain why no intact vegetative *Azolla* remains were found [7] and why the burial efficiency of organic carbon was found to be relatively low during this interval [31].

## Supporting Information

Figure S1 Minimum water temperature ( C±SE) in the top water layers and the bottom water layers of the freshwater and brackish water basins and the minimum air temperature (°C) at 10 cm above ground level during the mesocosm experiment.
(TIF)

Figure S2 Nutrient concentrations (µM ± standard error) in the freshwater and brackish water basins during the mesocosm experiment. **A**) Calcium concentrations, **B**) Magnesium concentrations, **C**) Sodium concentrations, **D**) Potassium concentrations and **E**) Sulphate concentrations in the top, middle and bottom water layers of the basins.
(TIF)

Figure S3 Oxygen concentrations (mg L^−1^±SE) in the top water layers and the bottom water layers of the freshwater and brackish water basins during the mesocosm experiment.
(TIF)
