# Supplementary figures and images for: Micro-Halocline Enabled Nutrient Recycling May Explain Extreme Azolla Event in the Eocene Arctic Ocean
Source: PLoS One. 2012 Nov 16;7(11):e50159. doi: 10.1371/journal.pone.0050159 (PMC3500341; doi:10.1371/journal.pone.0050159)

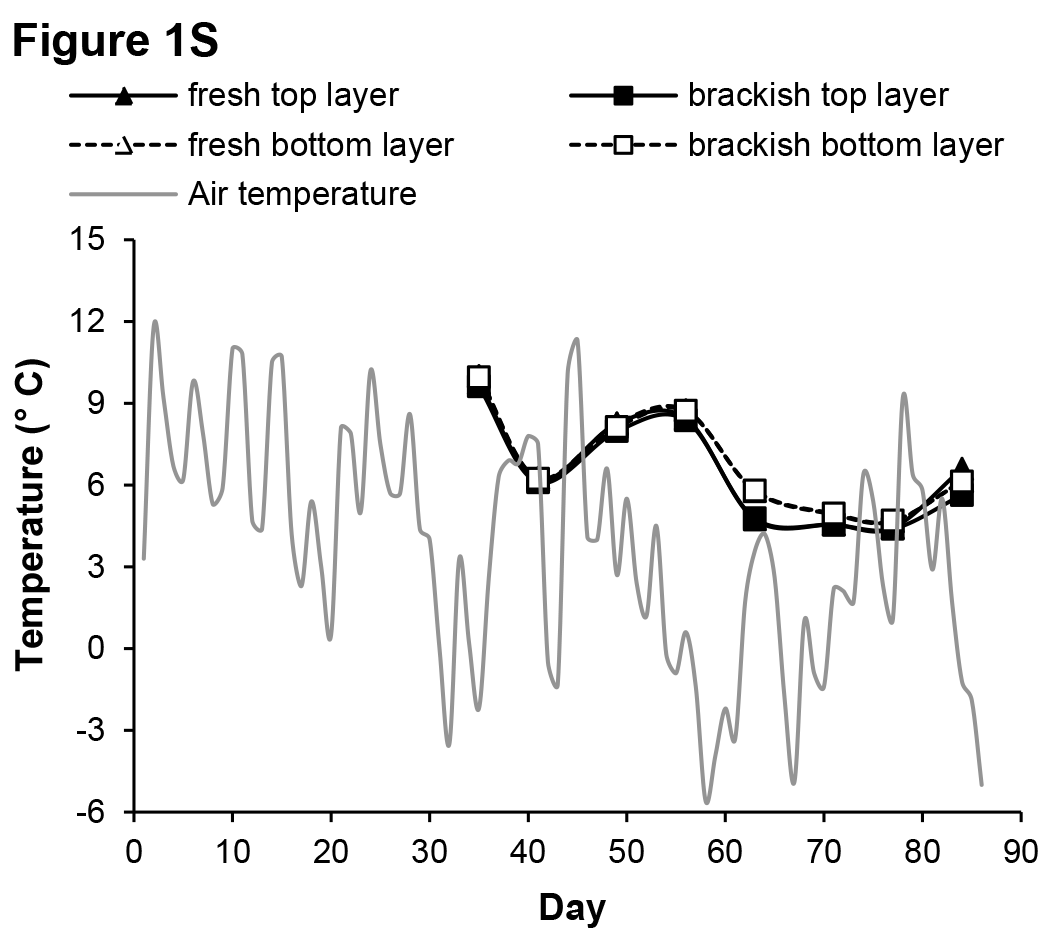

Supplement: Figure S1 — Minimum water temperature ( C±SE) in the top water layers and the bottom water layers of the freshwater and brackish water basins and the minimum air temperature (°C) at 10 cm above ground level during the mesocosm experiment. (TIF) [file pone.0050159.s001.tif]

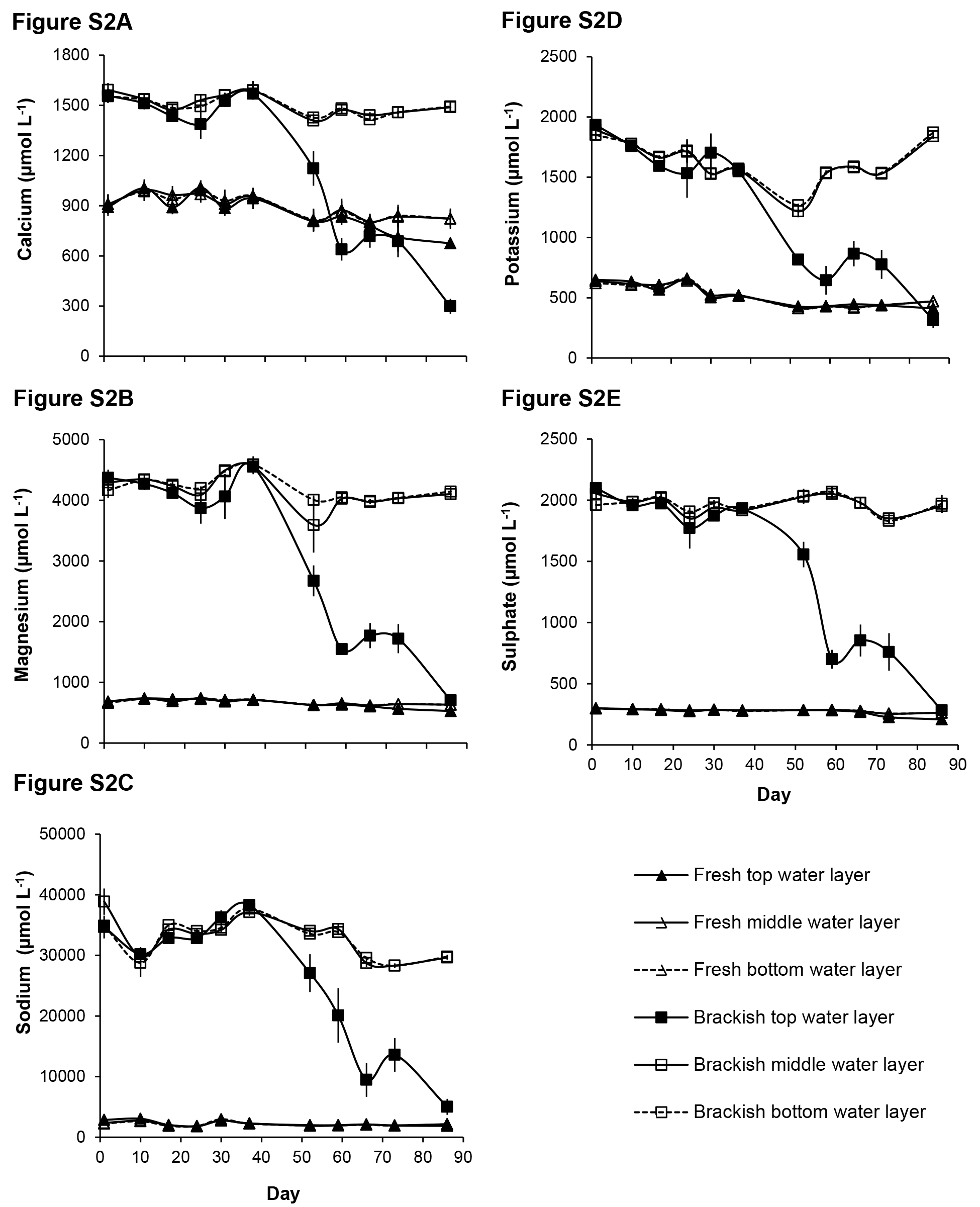

Supplement: Figure S2 — Nutrient concentrations (µM ± standard error) in the freshwater and brackish water basins during the mesocosm experiment. A) Calcium concentrations, B) Magnesium concentrations, C) Sodium concentrations, D) Potassium concentrations and E) Sulphate concentrations in the top, middle and bottom water layers of the basins. (TIF) [file pone.0050159.s002.tif]

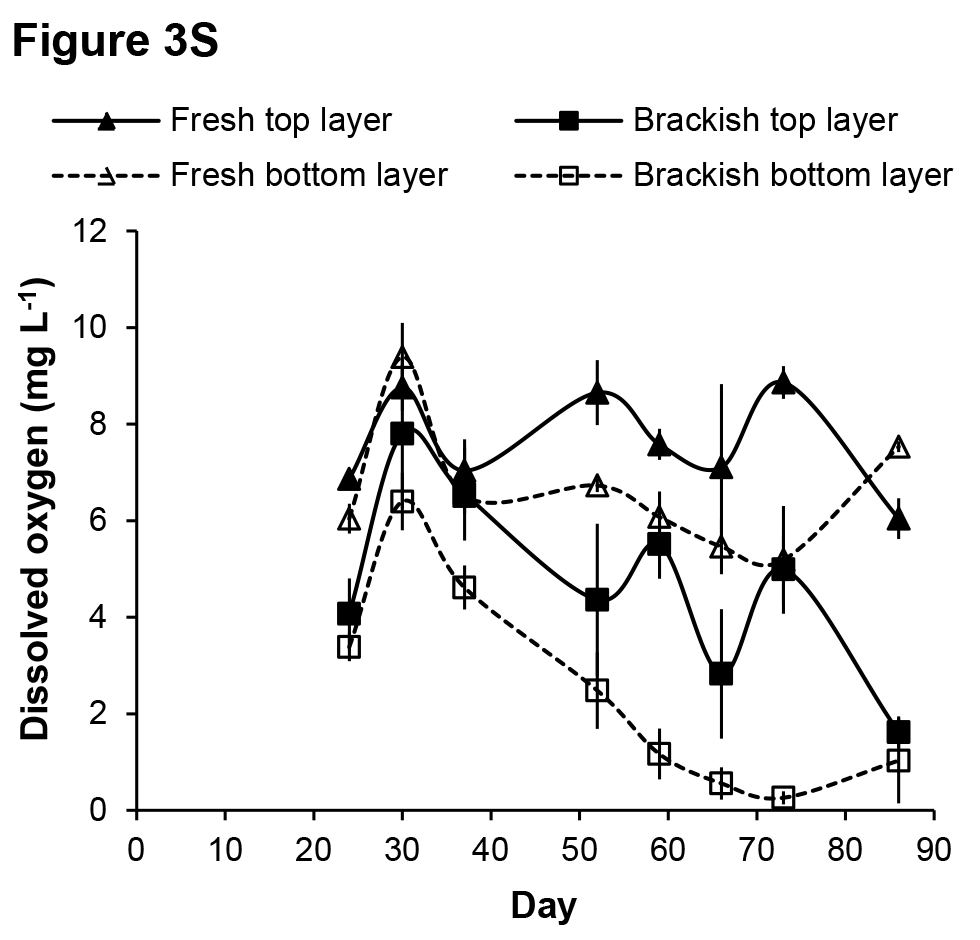

Supplement: Figure S3 — Oxygen concentrations (mg L−1±SE) in the top water layers and the bottom water layers of the freshwater and brackish water basins during the mesocosm experiment. (TIF) [file pone.0050159.s003.tif]
